# Validity and reliability study of the Turkish version of the Postoperative Recovery Index of patients undergoing surgical intervention

**DOI:** 10.3906/sag-1806-33

**Published:** 2019-04-18

**Authors:** Hande CENGİZ, Dilek AYGİN

**Affiliations:** 1 Department of Surgical Nursing, Faculty of Health Science, Sakarya University, Sakarya Turkey

**Keywords:** Surgery, patient care, postoperative care, recovery of functions

## Abstract

**Background/aim:**

For the purpose of providing the optimal postoperative care for patients and assisting them in terms of recovery, their health conditions and particular symptoms should be evaluated systematically with an appropriate measurement tool. This research was designed with the purpose of conducting the validity and reliability study of the Postoperative Recovery Index-Turkish Version (PoRI-TR) and determining the postoperative recovery conditions.

**Materials and methods:**

The sample of this study, which was planned methodologically and analytically, consisted of 382 patients who had a surgical intervention in a university hospital between September 2016 and June 2017. Analyses concerning the Turkish validity and reliability of the PoRI-TR were conducted. In the evaluation, a patient information form, the PoRI-TR, and the Quality of Recovery-40 Questionnaire (QoR-40) were used.

**Results:**

The PoRI-TR point average was calculated as 3.39 ± 0.916 and the Cronbach alpha reliability coefficient was calculated as α = 0.967. It was determined that the five-factor structure of the PoRI-TR, which was reduced from 37 items to 25, was adapted well.

**Conclusion:**

It was seen that the PoRI-TR is a valid and reliable measurement tool for Turkish patients.

## 1. Introduction

In the last years, the rate of complications after surgery has declined gradually due to advances in technology as well as developments in anesthesia, surgical and diagnostic techniques, and treatment. Therefore, surgical intervention has become the first method of choice [1,2]. It is predicted that with the help of these developments, early recovery can be achieved, and morbidity and mortality can be reduced along with prevention of postoperative stress response. The increased number of surgical interventions has also been influenced by the provision of quality care services and the increased level of importance attributed to health by individuals and society with increasing cultural levels [3–7]. Based on the surgical data of 56 countries among the 192 member states of the World Health Organization, it has been reported that approximately 234.2 million surgeries are performed annually [8]. 

Considering the Turkish literature on patients’ recovery after surgical interventions, there are a limited number of published studies. Karaman et al. [9] carried out the Turkish validity and reliability study of a measurement instrument that measures patients’ postoperative emotional states, physical comfort, patient support, physical independence, and pain-related recovery quality in 2014. On the other hand, psychological symptoms, physical activities, general symptoms, bowel symptoms, and appetite symptoms can be assessed up to 30 days after discharge by using the Postoperative Recovery Index (PoRI), the validity and reliability of which was tested by Butler et al. in 2012 [10]. This index is seen as a measurement tool that can be adapted to surgery types that are different from each other and can reflect versatile self-reports of the patient. Based on these necessities, we aimed to test the validity and reliability of the PoRI in patients undergoing surgical interventions.

## 2. Materials and methods

This study was planned methodologically and analytically to test the validity and reliability of the PoRI in patients undergoing surgical intervention. Before the study onset, written permission was received from Stephen F. Butler, who developed the PoRI, to test the validity and reliability of the Turkish PoRI scale. Institutional permission from the Sakarya Training and Research Hospital and ethics committee approval were then obtained from the Ethics Committee of Clinical Investigations of Sakarya University Faculty of Medicine (Ethics Committee Approval Number: 71522473/050.01.04/157, 09/01/2016). 

The research population consisted of 1648 patients who underwent surgical intervention (herniation surgery, thyroid-parathyroid surgery, cholecystectomy, and appendectomy) in the General Surgery Department of the Sakarya Training and Research Hospital at Sakarya University between September 2016 and June 2017. The sample of the study consisted of 382 patients from among those who met the inclusion criteria of the study and agreed to participate in the study. People who volunteered to participate in the study, were 18 or older, underwent surgical intervention, were not patients of oncologic surgery and trauma, did not use corticosteroid drugs, were not pregnant, and had no current major psychiatric diagnosis, no visual or hearing problems, no cognitive or mental problems, and no active systemic diseases were included within the sample group.

### 2.1. Data collection

The data were collected using a patient information form, the PoRI-TR, and the Quality of Recovery-40 Questionnaire (QoR-40). 

#### 2.1.1. Patient information form 

This form was prepared based on the literature [3,9,10]. It consisted of 22 questions including medical information such as age, sex, BMI, information about the surgery they underwent, and chronic diseases. 

#### 2.1.2. Postoperative Recovery Index (PoRI)

The PoRI that was tested for validity and reliability by Butler et al. in 2012 consists of 37 items. It has 5 subdimensions: psychological symptoms, physical activities, general symptoms, bowel symptoms, and appetite symptoms. Scores of the items included in the subdimensions are summed, their averages are calculated, and their subdimension scores are determined. For the PoRI total score, all of the 37 items are summed and their average is calculated. High scores received from the index reflect that more problems are experienced in postoperative recovery, whereas low scores show that postoperative recovery has been easier (Table 1) [10]. 

**Table 1:** Scoring system for the PoRI total score and subdimension scores.

No difficulty	1
Little difficulty	>1 to <1.5
Moderate difficulty	1.5 to <2.5
Severe difficulty	2.5 to <3.5
Extreme difficulty	3.5 to 5

#### 2.1.3. Quality of Recovery-40 Questionnaire (QoR-40)

The QoR-40 questionnaire was developed by Myles et al. in 2000 [11]. Its Turkish validity and reliability studies were carried out by Karaman et al. in 2014. The QoR-40 consists of 40 items. The scale has five subdimensions including emotional state, physical comfort, patient support, physical independence, and pain. Its items have scores ranging from 1 to 5 (5-point Likert scale). When determining subdimensional scores, the relevant items are collected. To obtain the total score, scores of all items are summed (the result of which ranges from 40 to 200). An increase in scores means that the physical and emotional well-being of patients are at an expected level after surgery. A low score means that the well-being is affected adversely [9]. Tests and analyses performed during the validity and reliability studies of the PoRI are summarized in Table 2.

**Table 2:** Technical and process transactions used for validity and reliability of the PoRI.

Validity technique	Transactions for validity technique
Content validity	Expert opinion
Criterion-dependent validity Peer-time validity	Correlation
Structure-concept validity Factor analysis	Confirmatory factor analysis Basic components analysis
Reliability technique	Procedures for reliability
Internal consistency Internal consistency coefficient	Cronbach alpha coefficient

### 2.2. Statistical analysis 

When assessing the data of the study, frequencies were provided for categorical variables, and descriptive statistics (means and standard deviations) were given for numerical variables. When investigating the difference between two categorical variables, the significance test of the difference between two averages (independent samples t-test) was used. When the number of groups was more than two, one-way analysis of variance (ANOVA) was used. Pearson’s correlation analysis was conducted to examine the relationship between two numerical variables. The number of patients (n) to be included in the sample of the study was calculated using the simple random sampling formula for cases where the population is known, and the number of patients undergoing minimal surgery was found to be 312. When the size of the sample was planned, in order to be able to carry out validity and reliability studies using factor analysis, the criterion that the sample size should be at least 5–10 times the number of items of a scale was taken into account, which is recommended in the statistical literature [12–14].

## 3. Results

Of the patients who were included in the study, 29.6% underwent cholecystectomy, 27.5% underwent herniorrhaphy, 26.4% underwent thyroid-parathyroid surgery, and 16.5% underwent appendectomy surgery. The average ages of females and males were 50.26 ± 14.63 and 52.34 ± 16.25 years, respectively. The body mass index of the patients was 27.76 ± 5.61. Of the patients, 55% were female and 45% were male. The patients spent on average 1.28 ± 0.829 days after surgery; 55% of them had undergone surgical intervention before. Of the patients, 87.4% were married, 79.6% were elementary school graduates, 45.8% were housewives, and 23.6% were retired. Moreover, 90.3% stated that their incomes balanced their expenses, 94% stated that they had social security, 72.3% were not smoking, 98.4% were not using alcohol, and 33.2% had chronic diseases.

### 3.1. Studies to ensure the language equivalence of the PoRI-TR 

In the first stage, studies on language validity were carried out in order to adapt the PoRI to Turkish patients and to determine its validity and reliability. First, permission was received from Stephen Butler via e-mail to translate and adapt the PoRI to Turkish. The back-translation technique was used to translate the original scale. In order to ensure the language equivalence of the scale, first, the index was translated by a researcher from English to Turkish. In the next stage, the scale was translated to Turkish by a total of five experts who had a good command of English and were native Turkish speakers, consisting of four university professors and one English language lecturer. The most appropriate expressions were selected by checking the suitability of the translated index for the original text, and the Turkish index was created. Next, the English language lecturer, who was a native Turkish speaker, was asked to translate the scale to English, without being shown the English version of the scale. The English translation and the expressions of the index were compared. The necessary corrections were made in the text, and it was shaped into its final form. Being shaped into its final form, the index was sent to Stephen Butler via e-mail, and his approval was obtained.

### 3.2. Studies carried out to ensure the validity and reliability of the PoRI-TR:

#### 3.2.1. Validity of PoRI-TR (Cronbach alpha reliability coefficient)

In this study, a Cronbach alpha value was calculated for each subdimension of the index. According to the results that were obtained, the Cronbach alpha coefficient of the PoRI-TR was α = 0.967. The Cronbach alpha coefficients of the PoRI-TR subdimensions varied between 0.93 and 0.983 (Table 3).

**Table 3:** Validity of Postoperative Recovery Index-TR.

	Cronbach’s alpha
Physical activities	0.978
Bowel symptoms	0.977
General symptoms	0.971
Appetite symptoms	0.983
Psychological symptoms	0.930
Postoperative Recovery Index-TR	0.967

#### 3.2.2. Content validity of PoRI-TR

During the language equivalence study, two methods were used: back translation (translation, retranslation) and comparison of the Turkish and English forms of the adapted index. A content validity index (CVI) was calculated for the PoRI-TR items that were sent to a total of 10 subject matter experts in the field of surgery to examine the content validity. Accordingly, it was seen that the CVI score of the PoRI-TR was 0.99, and the CVI scores of all items were greater than 0.80. In content validity, the average, standard deviation, maximum, and minimum scores given by the subject matter experts for the content validity of the PoRI-TR were examined. It was found that there was no difference between the expert opinions based on Kendall’s W concordance test for the content validity of the index (Kendall’s W = 0.070; P = 0.071) (Table 4). The relationship between the whole and subdimensions of the PoRI-TR and the QoR-40 scale was examined within the context of criterion-related validity. There was a negative moderate correlation between the average score of QoR-40 and the intestinal symptoms scores (P < 0.05). There were negative high correlations between the average score of QoR-40 and physical activities, general symptoms, request/desire symptoms, psychological symptoms, PoRI-TR total, physical comfort, emotional states, physical independence, patient support, and pain (P < 0.05).

**Table 4:** Kendall’s W concordance test.

	N	Mean	SD	Min	Max
Expert 1	25	4.00	0.000	4	4
Expert 2	25	4.00	0.000	4	4
Expert 3	25	3.88	0.332	3	4
Expert 4	25	3.88	0.332	3	4
Expert 5	25	3.88	0.332	3	4
Expert 6	25	3.88	0.332	3	4
Expert 7	25	3.68	0.627	2	4
Expert 8	25	3.92	0.277	3	4
Expert 9	25	3.92	0.277	3	4
Expert 10	25	4.00	0.000	3	4
Kendall’s W = 0.070			P = 0.071		

#### 3.2.3. Construct Validity of PoRI—exploratory factor analysis

The data of the study, which were collected from 382 people to identify whether the PoRI was compatible with Turkish, were transferred to the IBM SPSS Statistics 23 program. First, an exploratory factor analysis was run on this dataset, and the principal component analysis method was used to extract factors. No restrictions were set to limit the number of factors. Items with factor loads over 0.500 were allowed (Table 5). Therefore, based on the results of the factor analysis, the number of items dropped to 25, which had originally been 37. These 25 items were found to gather under 5 factors based on the content validity, and all factor loads were found to be over 0.500. The Kaiser–Meyer–Olkin (KMO) value was found to be 0.924 (Table 6). Thus, the factor analysis results to be applied on the data were found to be beneficial and useful. Bartlett’s test for sphericity revealed that there were significantly high correlations between the variables and that the data were suitable for a factor analysis [15] (c2(300) = 16841.176; P < 0.001). 

**Table 5:** PoRI-TR factor loadings.

	Factor loadings	Eigenvalue	Variance (%)
Physical activities		7.177	28.709
15. Walking up a flight of stairs	0.919		
14. Walking several blocks	0.915		
12. Getting yourself to the bathroom	0.874		
13. Dressing yourself	0.864		
11. Your ability to stand up	0.846		
16. Driving	0.836		
10. Your ability to sit up	0.820		
9. Doing your day-to-day activities (cleaning, working)	0.810		
Bowel symptoms		4.952	19.807
28. Feeling rectal pressure or fullness	0.936		
27. Bowel movements seem incomplete	0.927		
29. Bowel movements are unsatisfying	0.919		
26. Problem passing gas	0.894		
25. Gas pains	0.885		
Appetite symptoms		3.601	14.405
18. Only able to eat small amounts of food at one time	0.855		
19. Nothing tastes good (food or drink)	0.854		
17. Not able to enjoy my favorite foods	0.822		
20. Poor appetite	0.754		
General symptoms		3.585	14.340
36. Feeling not as productive as normal	0.842		
37. Motivation is low	0.840		
35. Worried that I won’t fully recover from my surgery	0.809		
34. Feeling discouraged	0.803		
Psychological symptoms		3.162	12.646
5. How often have you had trouble sleeping (falling or staying asleep)?	0.824		
4. How often have you had trouble staying awake during the day?	0.809		
6. How often have you had trouble focusing on mental tasks (e.g., reading, working crossword puzzles, following complicated directions)?	0.770		
7. How often have you noticed slurring your speech?	0.602		
Total			89.908

**Table 6:** The Kaiser–Meyer–Olkin (KMO) and Bartlett sphericity tests.

KMO test		0.924
Bartlett’s sphericity test	c2	16841.176
	SD	300
	P	0.000***

*:P < 0.05, **:P < 0.01, ***:P < 0.001.

The subdimensions of physical activities, bowel symptoms, general symptoms, appetite symptoms, and psychological symptoms explained 28.709%, 19.807%, 14.405%, 14.340%, and 12.646% of the total variance, respectively. These five factors accounted for 89.908% of the total variance. 

#### 3.2.4. Confirmatory factor analysis (PoRI-TR)

The confirmatory factor analysis was run on the dataset with 382 cases in the IBM SPSS Amos 22 program. In the first stage, a first-order confirmatory factor analysis (CFA) model was created as shown in the Figure. In this model, 5 factors/dimensions (F1: physical activities, F2: bowel symptoms, F3: general symptoms, F4: appetite symptoms, and F5: psychological symptoms) were included as latent variables and the expressions forming these factors were included as observed (indicator) variables. In the second stage, the maximum likelihood method was used, which is frequently used in structural equation models and gives reliable results even in cases where the data are not normally distributed. It was aimed to predict the parameters including the errors of the observed variables, the variances of the latent variables, and the regression coefficients of the paths drawn to the observed variables from the latent variables. In order to improve the fit indices, two-way relationships were established between the error terms of the following pairs of questions, which had the highest fit index values in PoRI: “10. Your ability to sit up” - “11. Your ability to stand up” - “25. Gas pains” - “26. Problem passing of gas” “34. Feeling discouraged” - “35. Worried that I won’t fully recover from my surgery”. Moreover, relational hypotheses were established between dimensions to determine the expected covariance between dimensions, and the relationships between dimensions are also shown in the Figure.

**Figure 1:**
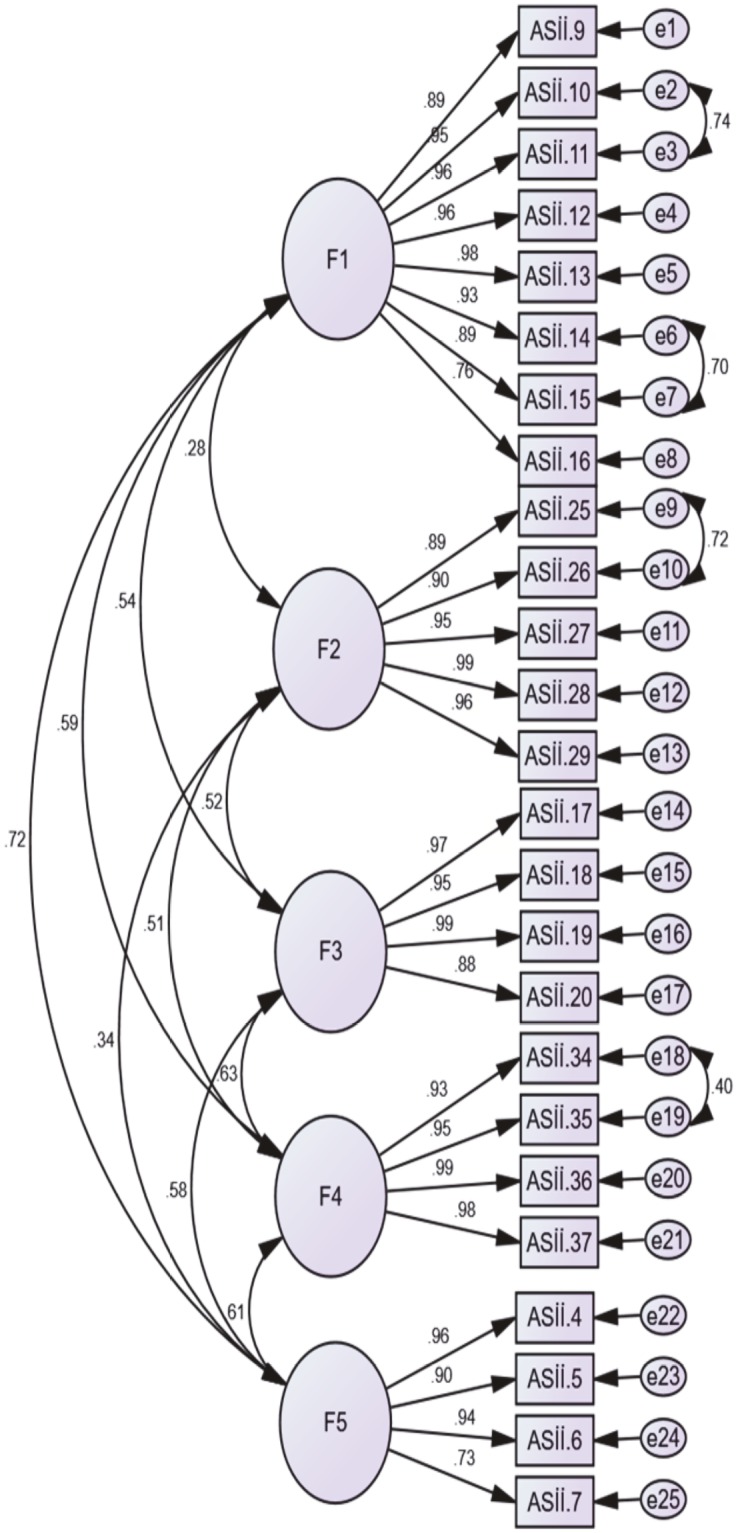
First-order CFA model.

In the final stage, the fit indices of the first-order CFA model created with 5 dimensions were examined. According to the research findings, the five-factor structure of the PoRI-TR with 25 items had a good fit in general. Additionally, the Figure also shows the relationships between the subdimensions.

Considering the fit index values, the values of χ2/df (chi-square/degrees of freedom), IFI (incremental fit index), TLI-NNFI (nonnormed fit index), CFI (comparative fit index), and SRMR (standardized root-mean square residual) were all good (Table 7).****

**Table 7:** Fit indices of the CFA model.

χ2/df	GFI	IFI	TLI	CFI	RMSEA	SRMR
4.914	0.797	0.940	0.931	0.940	0.101	0.0467

## 4. Discussion

It was concluded that the PoRI-TR was suitable for the Turkish language and culture in terms of language equivalence. Regarding the reliability of the PoRI-TR, the alpha coefficients of the whole index and its subdimensions were found to be in the range of 0.80 ≤ α < 1.00. The alpha coefficient is a weighted standard deviation average, which is calculated by dividing the total variance of ‘k’ items in the scale by the overall variance, and the generally accepted value of the coefficient is at least 0.70 [16,17]. The higher the coefficient, the higher the reliability (if 0.00 ≤ α < 0.40, the scale is not reliable; 0.40 ≤ α < 0.60, scale reliability is low; 0.60 ≤ α < 0.80, the scale is quite reliable; 0.80 ≤ α < 1.00, scale reliability is high) [18]. Therefore, as indicated in the literature, the PoRI-TR is a highly reliable measurement tool.

Many techniques are used for the evaluations done by the subject matter experts on content validity, although generally the CVI and Lawshe’s and Davis’s techniques are used [13]. In Davis’s technique, the ratings of items are in the form of “appropriate (a)”, “the item should be revised slightly (b)”, “the item should be revised substantially (c)”, and “the item is not appropriate (d)”. In Davis’s technique, an item’s CVI is calculated by dividing the number of experts who selected (a) and (b) by the total number of experts, and when the value obtained is equal to or greater than 0.80, that item is considered to have an acceptable level of content validity [17,19]. It was found that the CVI score of the PoRI-TR was 0.99, and the CVI scores of all items were greater than 0.80; therefore, the PoRI-TR had content validity.

Criterion-related validity or concurrent validity is also known as “similar scale validity” or “synchronous scale validity”. According to this approach, two separate measurements of the same concept are compared at the same time point (at the same time, or at the closest time) [17,20]. In line with this information, the PoRI-TR and QoR-40 questionnaire were administered at the same time, and the concurrent validity was tested. Considering the correlation between the PoRI-TR total scores and the QoR-40 total scores of the 382 patients, it was seen that there was a significantly negative high correlation. Our results showed us that PoRI-TR had criterion-related validity.

In this study, for construct validity, Bartlett and KMO tests were used to determine whether the PoRI-TR data were suitable for factor analysis. The KMO test is a feasibility test to check the presence of correlation between variables and whether factor analysis is appropriate. According to the results of the KMO test, the values ranged from 0 to 1. It has been stated that if this value is less than 0.50, the test is “unacceptable”; 0.50–0.60 is bad, 0.61–0.70 is weak, 0.71–0.80 is medium, 0.81–0.90 is good, and values above 0.90, are perfect [21]. In this study, the KMO test value of the scale was found to be 0.924. Because this value that was obtained can be interpreted as perfect, the measurement results obtained from the PoRI-TR were found suitable for principal components analysis. According to the Bartlett test for sphericity, it was concluded that there was a statistically high level of correlation between the PoRI-TR variables and that the PoRI-TR data were suitable for factor analysis (c2(300) = 16841.176; P < 0.001).

According to the exploratory factor analysis that was carried out, the higher the percentage of variance, the stronger the factor structure of the scale that was developed/tested for validity. Eigenvalues are the sums of the squares of factor loads. If these totals are greater than 1 for each subdimension, it means the questions that are collected under that subdimension together are sufficient to explain that subdimension [11].

According to the results of the CFA that was carried out in this study, a first-order CFA model was created in the first stage. In the second stage, when the model was predicted, the maximum likelihood method was used. Moreover, relational hypotheses were established between dimensions to determine the expected covariance between dimensions. The “goodness of fit” statistics need to be above a certain level to ensure the construct validity of a scale within the scope of CFA [13]. When the fit values obtained from this study were considered in light of the literature [22–24], it was seen that the χ2/df, IFI, TLI, CFI, and SRMR values were good, but the GFI and RMSEA values were unacceptable. When the fit indices were considered in general, it was concluded that the PoRI-TR was acceptable. In this context, the five-factor structure of the PoRI-TR that included 25 items overall had a good fit. 

In conclusion, this study proved that the Turkish version of the PoRI was a valid and reliable instrument to measure postoperative recovery in Turkish patients. The PoRI can be used to measure postoperative recovery. The PoRI-TR can be administered by itself or in combination with other scales for relevant patients if it is deemed suitable. In this research study, because the PoRI-TR was administered to certain diagnosis groups, it is recommended to administer the scale for different types of surgical interventions in order to contribute to the literature. Moreover, carrying out studies with larger samples and well-designed methodologies, and collecting the results of such studies within a common database, will ensure that we have a collection of reliable statistics corresponding to our country and culture. The spreading of such studies will also encourage the development of different scales and their adaptation to our society.
